# A Letter to the Editor of the London Medical and Physical Journal, in Reply to a Letter from Mr. Dickinson to Dr. Grattan

**Published:** 1821-03

**Authors:** Richard Grattan

**Affiliations:** Fellow and Censor of the King's and Queen's College of Physicians in Ireland; Physician to the Fever Hospital and House of Recovery, Dublin; &c.


THE LONDON

### Medical and Physical Journal.

### Original Communications, Select Observations, etc.

---

*A Letter to the Editor of the London Medical and Physical Journal, in Reply to a Letter from Mr. Dickinson to Dr. Grattan.* By RICHARD GRATTAN, M.D. Fellow and Censor of the King's and Queen's College of Physicians in Ireland; Physician to the Fever Hospital and House of Recovery, Dublin; &c.

"Let us be judged, both by the public and among ourselves, rather by our works than by our own conceits."—DICKINSON'S *Letter to* Dr. GRATTAN.

SIR,

HOWEVER reluctant I may be to engage in controversy, I cannot avoid troubling you on the present occasion, in answer to a Letter addressed to me in the last Number of your Journal. I confess that I am not displeased to find that my publication on the "State of Medical Practice in Ireland," should have attracted the attention of the profession in England; and I am particularly gratified to perceive that you have considered the subject of sufficient importance to devote so large a portion of your Journal to the insertion of the observations of Mr. Dickinson in reply to my "Remarks."

In assuming to myself the merit of purity of intention, and of a wish to support the respectability of my profession, uninfluenced by illiberal or selfish considerations, I willingly ascribe to Mr. Dickinson motives equally honourable and conscientious. I have no doubt but that his object is to arrive at the truth, and to assist others in forming a right opinion on a matter of such moment as the improvement of medical practice, in the proper regulation of the different departments of which every member of society is interested. I shall therefore, without further apology, proceed to examine his statements, and endeavour to set him right as to some points, on which I consider him to be altogether in error.

However attentively he may have perused my essay, he has, in several instances, (I am sure unintentionally,) totally misrepresented my meaning; and, like Don Quixotte in his furious assault on the barber and his basin, suffered his imagination so far to prevail over his judgment, as to create an opposition for the sole purpose of appearing to overcome it. It cannot be expected that I should now occupy my time in correcting Mr. D.'s misconceptions. Perhaps, a more attentive perusal of my "Remarks" may enable him to discover them: at least, I believe the majority of my readers are not likely to fall into similar mistakes. A great portion of Mr. Dickinson's letter I shall, therefore, pass over in silence; although, in so doing, I must be understood not as assenting to his positions, but rather as considering them too unimportant to merit a particular refutation.

The object which I had in view in publishing my "Remarks," was to show the necessity of preserving the different branches of the medical profession as distinct from each other as possible. Convinced of the expediency of this measure, and of the advantages which the public must derive from its general adoption in cities and in populous districts, I argued against the admission into the College of Physicians of practising surgeons and apothecaries; and, in the second part of my "Remarks," suggested a plan which appeared to me to obviate every objection that could be advanced against the practicability of the measure which I supported.

Mr. Dickinson, on the contrary, thinks that the public interests are best promoted by encouraging the class of "general practitioners," or those individuals who practise the three branches indiscriminately. On this point we are completely at issue. I have already explained my reasons for contending that the physician should alone be considered competent to treat those diseases which by universal consent are termed medical; and the names and leading symptoms of the greater number of which, Mr. D. will find by referring to "Cullen's First Lines of the Practice of Physic." When I use the term physician, I do not mean, as Mr. D. would seem to insinuate, "men dubbed doctors without a thorough knowledge of the healing art, merely in consequence of a collegiate residence and academic accomplishments," but physicians practically conversant with their profession, and who have super-added to their "academic accomplishments," which Mr. D. affects to despise, an acquaintance with disease to the full as extensive as can be ascribed to any surgeon or apothecary by the most zealous advocate for either.

4

That "some diseases are treated principally by regimen and internal remedies, while others, on the contrary, are managed for the most part by mechanical means,"* Mr. D., I believe, will not be disposed to deny; and I can hardly persuade myself he seriously thinks that, differing as they do in their treatment, the same system of study is equally calculated for physicians and surgeons. To the physician belong all those diseases which affect the entire system, and to the surgeon those which relate to fractures and dislocations, to wounds and to operations. Surely, no one who pretends to possess the slightest portion of common sense would affect to believe, or endeavour to persuade others, that, because surgical diseases are often accompanied by constitutional symptoms, and medical diseases by local complaints, the entire should be confounded, and this distinction of diseases so obviously different, termed "an arbitrary and unnatural division;" that, because "it is impossible to define their limits," and "a bond of connection is so intimately established between all the parts of the living system," these circumstances should be represented as sufficient "to defy every attempt to consider diseases separately and by piecemeal, general or local, internal or external, exclusively." As well might Mr. D. assert that morning and evening are the same, and that any distinction between them is "arbitrary and unnatural," because he cannot determine the precise moment at which twilight begins and ends.

Common sense and the experience of ages, however, are so opposed to the opinions of Mr. D. that it is almost unnecessary to dwell on the subject of separating medicine from surgery; and therefore, instead of entering into physiological discussions, or repeating what I have already mentioned in my "Remarks," I would simply ask whether, in the case of a patient affected with a calculus in the bladder, if an operation were considered inadmissible, the physician, conversant with chemistry,—capable of determining experimentally the proper solvent of the calculus,—of ordering the medicines adapted to prevent its increase, and the means calculated to allay irritation,—would not be more competent to treat the disease than a surgeon full of all the knowledge of the dissecting-room? And, on the other hand, if the constitutional symptoms and other circumstances rendered an operation advisable, would not the surgeon then supersede the physician with advantage to the patient? I would ask, whether the physician who devotes his entire attention to the treatment of any particular disease,—be it fever, dropsy, gout, or consumption,—must not, from his extensive practice and constant habit of considering the symptoms of such disease, as they occur under different circumstances and in different individuals, attain a more perfect knowledge of the nature of the disease, in its different shades and varieties, than if he were only to meet it occasionally in the ordinary routine of common practice? I would ask, must not the surgeon, who operates for the stone exclusively,—who has devoted his entire attention to ascertain the comparative advantages and disadvantages of the various methods of operating, and the circumstances under which one kind of operation is to be preferred to another,—be supposed to operate with more success than if, professing the entire art of surgery, he were to draw teeth, bleed, cup, operate for artificial pupil, amputate, and trepan, in succession? To this there can be but one answer; and yet Mr. D. asserts, "that some of our most eminent surgeons, considering it impossible to form a line of separation, either on the principle of science or on the ground of public good, practise not only surgery in all its branches, but medicine also: and," he adds, modestly enough, "both of the departments with the greatest honour to themselves and advantage to society."

That Mr. D. and some other "eminent surgeons" should advance such opinions, is not at all wonderful: the question is, are the opinions which they entertain warranted by reason and experience? That they are not, I am persuaded; nor am I singular in thus thinking; and, although I am not in general disposed to rest my arguments on the mere authority of any individual, yet, were I to decide on such ground, that of Mr. Abernethy, I contend, is an authority more than sufficient. Mr. Abernethy, a surgeon of as much talent, and at least as eminent, as any of those on whose judgment Mr. D. relies, in the Hunterian Oration delivered by him in 1819, before the London College of Surgeons, which I quoted in my "Remarks," states it as his opinion, "that the division of medicine into two great departments, which custom has established, seems also to have received the fullest sanction of experience; and, were we not to acquiesce in it, *we should subvert the institutions of society, and throw the whole profession into confusion.* So much, also, is to be known and done in either department, that, if we invade each other's province, we must neglect properly to cultivate and improve our own."

Admitting that Mr. Abernethy formerly entertained different sentiments, this very circumstance is, I think, a strong argument in support of the correctness of those which he now adopts, as it places in a most respectable light his candour and sincerity, and affords the most decisive testimony of his anxiety to discover truth, and of his readiness to acknowledge and to adopt it.

All the remarks of Mr. D. relative to my opinions as to minute and morbid anatomy, are totally inapplicable; for I no where asserted that to the physician dissection was unnecessary. I distinctly stated the importance of anatomy, so far as it is applicable to medical purposes; but intimated that its minutiæ, such as the exact course of every nerve and artery, and the origin and insertion of every muscle, were in a great measure superfluous, and that such knowledge should not be obtained at the expense of more useful information. On the subject of anatomy, either minute or morbid, Mr. Dickinson and I, I fear, are not likely to agree; which I regret the more as I presume it is "a favourite pursuit," if an opinion may be formed from the—I will not say "vivid style," but ardent manner in which he speaks of its importance. In his anxiety to correct my errors on this head, after informing me "that every thing we understand concerning the living principle is entirely derived from our knowledge of the phenomena resulting from function," he immediately observes, "that all our knowledge of the organs, and the manner in which their functions are performed, is solely derived from patient research into the mechanism of animal structure;" a position from which I most distinctly and decidedly dissent,—which I pronounce to be erroneous in theory, and dangerous in practice,—which leads me to think that Mr. D., notwithstanding his anatomical arguments, cannot be acquainted even with the first principles of physiology, and convinces me that the practitioner who values himself on his imagined anatomical superiority "will form only an ill-educated, half-taught, incompetent, and therefore a dangerous, physician."

In support of the pretensions of surgeons and apothecaries to be admitted as licentiates of our College, Mr. D. mentions that, "if you refuse the solicitation of a candidate to substantiate his claim to the honour he seeks by a demonstration of his fitness, let the measure be adopted when or where it may, the motive which directs the refusal is founded on sinister views, inseparable from the selfishness and illiberality which you so distinctly disclaim." "Perhaps the soundness of policy to which you look up, may have a character distinct from the general good; and the regulations you are so determined to maintain, may be unworthy to exist."

Mr. D. writes on a subject which he does not seem to understand. Does he know why the College of Physicians was incorporated; and is he aware of the duties which its members have to perform? In the preamble to our charter it is stated, "that King Charles the Second, duly considering the daily abuses of the most laudable and necessary art of physic in the kingdom of Ireland, by the practice of mountebanks, empirics, and other ignorant and illiterate persons, to the impairing and hazard of the lives of his good subjects, did, for the remedy of these and other mischiefs, and for the encouragement of the learned and experienced practitioners in physic, and for the benefit and safety of his good subjects, by letters patent, found and establish a college or corporation of physicians in the city of Dublin:" that, "because their power and jurisdiction did not extend further than seven miles from Dublin, all the rest of the kingdom was exposed to the same inconveniency it was liable unto before the said grant; whereby the number of unskilful and illiterate practisers of physic hath much increased, and the frauds and deceits of empirics, apothecaries, and druggists, doth abound, to the dishonour of our government, and to the great prejudice and destruction of our good subjects. For the remedy, therefore, of those and the like evils, and for the prevention of the like mischiefs for the time to come, and to supply the defects of the former charter," their present charter was granted to the College by William and Mary.

Does he know why the College of Surgeons in Ireland was founded; and why the Company of Apothecaries, also, was established? Is it not evident that the legislature appointed each corporation, to prevent, as far as possible, abuses in their own department? thus recognizing the propriety of rendering each department distinct; and, of course, imposing on the College of Physicians, from its more extensive powers, the duty of carrying its intention into effect, and of preserving them separate. Is Mr. D. aware that in Ireland the law requires an apothecary to be apprenticed for seven years, before he can be examined as a master; that, in the College of Surgeons, an apprenticeship of five years is necessary; and that, in our University, the degree of M. B. is not conferred until seven years after the period of entrance? I cannot imagine that he is ignorant of these facts; nor can I persuade myself that he considers such precautions for the public good as "inseparable from selfishness and illiberality."

Mr. Dickinson evidently is not a close reasoner. His arguments in general either prove nothing, or, what is worse, they prove too much for his purposes. I shall endeavour to reduce his reasoning to a syllogistic form, and ascertain to what sort of conclusion it will lead. He says,

"If you refuse the solicitation of a candidate to substantiate his claim by a demonstration of his fitness, the motive which directs the refusal is inseparable from selfishness and illiberality.

"But, a candidate may be fit who has not been previously apprenticed, or regularly educated.

"Therefore, to refuse the solicitation of a candidate who has not been apprenticed or regularly educated, is inseparable from selfishness and illiberality."

If such be really the opinion of Mr. D., he indeed belongs to that class of persons who, to use the words of Mr. Abernethy, "would subvert the institutions of society." According to his view, every one, "by a demonstration of his fitness," would have a right to obtrude himself where it would be a violation of propriety to admit him, and an insult to common sense to suppose that he ought to be admitted. A tailor or a shoemaker might be a more pious character, a better biblical scholar, and more deeply read in works on divinity, than the Archbishop of Canterbury or of Dublin; and he might therefore wish, by "demonstration of his fitness," to substantiate his claim to promotion in the church: yet I can well conceive that the motive which would refuse to allow him to do so, is neither "selfish nor illiberal;" and this, plainly, because his previous habits and ordinary avocations are altogether incompatible with the functions of the high office to which he would aspire. Mr. D. surely must know that an attorney is not permitted to practise as a lawyer; and yet I have never heard it urged, as a reproach to the bar, that such a regulation was selfish or illiberal. The College of Surgeons in Dublin refuse to acknowledge, and strike from their list, any of their members who practise as apothecaries: they refuse to consult with such surgeons as are not members of their own body; and would object to recognize either Mr. Abernethy or Mr. Astley Cooper, or even Mr. Dickinson himself, were he to settle in Dublin. They opposed, and successfully resisted, a Bill which was lately attempted to be passed, for the purpose of rendering members of the London College capable of holding situations in Ireland: and is Mr. D. therefore prepared to charge his own profession with "selfishness and illiberality?" Does he mean, because they are so tenacious of their own privileges, to represent their policy as having "a character distinct from the general good?"

In London, the College of Physicians require that a candidate for their license, who has been formerly either a surgeon or apothecary, shall, before his admission to an examination, procure himself to be disfranchised by the body to which he previously belonged. Does Mr. Dickinson mean to charge the London College of Physicians with illiberality? or, if the usage of the London College be correct, why should the Dublin College be less careful of their reputation, or less attentive to the public interests; and why should that policy which is proper in London be disapproved of in Dublin, and the regulations necessary to uphold it designated as "unworthy to exist?"

Mr D. does not always clearly state his own arguments, and therefore I am not surprised that he should either misconceive my sentiments or express them obscurely. To a reader of his letter who had not previously perused my "Remarks," it would appear that I resisted the admission, on any terms, of surgeons or apothecaries into the College of Physicians;—that, because an individual happened to have been at any former period a surgeon or apothecary, I wished to confine him for life to his original "cast," and for ever deprive him of the power of changing it for the profession of the physician. On the contrary, I distinctly recommended that the example of the London College should be followed, which, "while it violates no principle, and exacts nothing harsh or oppressive, is found sufficient to answer the proposed end. It does not go so far as to confine such persons for ever to the business which they first professed, or tend to render them incapable of participating in the honours and advantages which the physician enjoys. A resolution of this kind would be illiberal and unjust: but the practice of the London College is not liable to this charge; it imposes no unfair restraint; it only places them on a level with other physicians, by obliging them to renounce their former profession. In this there evidently is no hardship. If the art of the apothecary be less profitable or less respectable than the profession of medicine, there can be no hardship in resigning it for one more profitable and more respectable. If, on the contrary, his business possess superior advantages, and if the apothecary do not wish to give it up in order to become a physician, his continuing to practise pharmacy is altogether a matter of his own choice."*

But, Mr. Dickinson may reply, the London College have two classes of licentiates,—those who practise in London, and those who reside in the country-parts of England. Admitting that medicine and surgery ought to be separated in "cities," why refuse to license as physicians those surgeons and apothecaries who practise in the country? You yourself, in the second part of your "Remarks," allow that, under certain circumstances, it may be necessary for the same individual to combine in his own person the different branches of the profession. To this I answer in the words of the "Address" from the Licentiates to the College of Physicians, quoted in my "Remarks." "They [the licentiates] would further suggest, that, although in the country parts of this kingdom it may be necessary to combine the different branches of medicine, yet this, being essentially a defect, ought not, on any account, to receive the sanction of the College, as such sanction would be in effect a sacrifice of principle, and an avowal that the propriety of distinguishing the professions was at least questionable."

---

Besides, as there is no law to prevent an apothecary or surgeon from practising medicine, a license from the College of Physicians can be only useful by enabling the public to distinguish between the comparative pretensions of different individuals, so as to dispose them to give a preference to the regularly-educated practitioner. But in country districts, which only support a single practitioner, such competition cannot exist. The practitioner, no matter what his medical qualifications may be, will practise medicine, whether licensed or not; his own necessities, and the exigencies of the country in which he resides, requiring him to do so. For what purpose, then, should he be licensed by the College? or how would the community be benefited by such a measure? If, however, a regular practitioner were to reside in the same neighbourhood, why should the surgeon and apothecary, each of whom possesses privileges peculiar to himself, be placed on a level with the physician, by an act of his own College;—a college "established for the management of the learned and experienced practitioners in physic?" Mr. D. says, "None, I think, can refuse their general assent to the physician's skill;" but, if the College of Physicians are further persuaded that physicians are more competent to treat diseases than surgeons or apothecaries, how could they, without a breach of their duty to the public, license the latter, perhaps to the total exclusion of the physician?

I now pass to the subject of confining apothecaries to their shops, and restricting them entirely to the preparation and composition of medicines. Mr. Dickinson remarks, "If the Irish apothecaries and others, whom you specify to be thus ignorant and incapable, deserve to rank with their fraternity in England in their elementary education and professional attainments, which I should take to be the case, from the statement you make of their growing influence with the public, your invectives against them are surely illiberal, and the ground of your aspersions wholly untenable."

Whatever difference of opinion may exist,—however sceptical some may be as to the utility of rendering medicine distinct from surgery,—I cannot conceive how it is possible for any one to contend that an apothecary should be permitted, much less sanctioned and encouraged, to neglect his shop, and to leave the preparation of his medicines and the compounding of the recipes of physicians and surgeons, to illiterate shopmen or careless and ignorant apprentices. In my "Remarks," I stated at length my reasons for resisting, "in every possible way," the admission of practising apothecaries as licentiates of the College. I contend that, were the College to give their license to a practising apothecary, however extensive his information or superior his attainments in medicine, they would deserve to forfeit their charter: and I avow that I should myself be one of the first to apply for such disfranchisement, on the ground of their having betrayed the interests of the public and of the profession.

On the "elementary education and professional attainments" of the "fraternity of England," I shall not offer an opinion. In my publication I described matters as they exist in Ireland; and I stated that, "according to the present system, the apothecary, by whom the medicines prescribed by the physician are presumed to be prepared, is scarcely ever in his shop. The moment he becomes a master, he in fact ceases to be an apothecary. From that moment he considers himself a medical practitioner, and regards the business of the apothecary as quite a secondary pursuit. He procures a mere school-boy as an apprentice, and to him is entrusted the serious, the important, office of compounding medicines. The most active poisons are placed within his reach,—the tinctures of opium and digitalis, extracts of hemlock and henbane, the arsenical solution, and the caustic alkalies, &c. are all at his disposal.

"These medicines may, and have often been confounded with others of much less activity, and have thus been administered in doses sufficient to destroy life. We know that such accidents do occur,—the daily prints constantly inform us of them; and yet, so powerful is the influence, they scarcely produce any effect on us. Accustomed to hear of such occurrences, we consider them as mere matters of course; and, finding that we have not ourselves suffered, we never anticipate the possibility of danger. Confidence does not impart security, though habit may totally suppress every feeling of apprehension. On the side of Vesuvius, over which torrents of lava have perhaps a hundred times flowed, the proprietor cultivates his vineyard, undisturbed by the vestiges of former ruin and devastation which every-where surround him: but is he on that account the more secure? Hazardous as his situation may be, it is not more so than that of the patient whose medicines are prepared by an ignorant or giddy apprentice, entrusted with the possession of active remedies. The public are not aware of the danger to which they are exposed from this cause. Physicians write their recipes in a dead language; in the hurry of prescribing they frequently use signs and abbreviations, and do not at all times write even these distinctly. The difference between the mark for a drachm and an ounce is trifling, and requires a practised eye to distinguish it at once. Sometimes in the names of very different medicines only a trifling difference exists.

"The legislature requires that an apprenticeship of seven years shall be devoted to the craft and mystery of an apothecary; but now a raw school-boy, in his master's absence, mixes and compounds at his discretion. A recipe is handed to him, which he is probably incapable of reading, and which he is told must be prepared with the greatest expedition. Is it to be supposed that the apprentice will hesitate, confess his ignorance, and wait until his master's return? No such avowal, I will venture to say, is ever made. The medicine must be prepared at all hazards; and accordingly the ingredients in a remedy, on the efficacy of which a patient's life may depend, become a matter of conjecture, instead of being accurately and faithfully compounded.

"When medicines are substituted for those which the physician intended, and effects different from what he had anticipated are produced, his situation is rendered most arduous. He is completely misled: he naturally supposes that he has taken a wrong view of the disease; he is deterred from persevering in the remedies which he before considered useful, and he, perhaps, has recourse to others not at all suited to the real nature of the complaint. These are points which deserve the most serious consideration. These facts alone are sufficient to convince every reflecting mind, that the master apothecary ought on no account to neglect his shop, and confide it to the care of his apprentice."*

These also are reasons sufficient to cause us indignantly to spurn the imputation of being actuated by "other motives than the justice of the cause;" and they are reasons which, I think, should have induced a liberal and candid writer to pause before he advanced such an insinuation. Does Mr. D. really believe that my statements, or my "aspersions," as he terms them, are the result merely of "peevish discontent," and that they have no other foundation than the disordered fancy of a prejudiced and dissatisfied mind? Or does he think that I am singular in my opinions; and is he sincere in doubting that such abuses can exist in the department of pharmacy? If he is still incredulous, I can adduce an authority in corroboration of my assertions, which may perhaps have greater weight with him. The celebrated Dr. Lucas, who formerly represented the city of Dublin in Parliament, was originally an apothecary. While he practised as such, he published his "Pharmacomastix, or the Office, Use, and Abuse, of Apothecaries explained." In this work, which was printed in the year 1741, and addressed to a member of Parliament, with a view to obtain an Act for the better regulation of the profession of the apothecary, he says, "It is not to be reasonably imagined that I should know-

* *Remarks*, pages 50, 52.

ingly do any thing to the prejudice of a profession, which I have made my choice, by which I have hitherto lived, and still propose to follow." "I will, however, confess that I have some *selfish private* (though not altogether mercenary) *views*, in soliciting a reformation of pharmacy, and a strict examination of apothecaries and their shops; because I would from myself, as well as others, remove all temptations to the abuse or corruption of my profession."

"Nothing assuredly can contribute more to the improvement and benefit of the healing art, than the professors of the different branches thereof applying themselves entirely to the study and practice of their respective callings. And this was certainly the wise design of our predecessors, in dividing it into those three several parts; and, were it yet further subdivided, it is probable it might be brought to greater certainty, and rendered more generally beneficial to mankind, than it can be in an aggregate state; since the meanest branch can sufficiently employ the whole attention and understanding of a man. We find that many of the ancients were of this opinion, and that it was approved of by the late celebrated Dr. Harris, of London. What considerable discoveries and improvements have hitherto been made in physic in general by these divisions, are very obvious to all that are conversant with the history of medicine. Physicians, by appointing proper agents for the more operose and mechanic part of their profession, threw off all incumbrance, and obtained more time and leisure for study. Chirurgery was cultivated under the same management; and both have arrived at the extraordinary pitch of eminence we now see them in, under these wise regulations. Pharmacy, too, while it kept within the proper bounds, shone and flourished; for it is observable that no set of men made a better figure in their way, than the apothecaries that retained their integrity and kept within the just limits of their occupation; which the works of many of them amply testify."

Having enumerated the names and works of several writers on pharmaceutical subjects, he observes, "While pharmacy was practised by such able hands as those, physic, which has so great a dependence upon it, must have flourished. But such became the insatiable avarice of most of the apothecaries, that they could not long content themselves with the ample profit that arose upon the sale of their proper commodities and manufactures; but, envious of their elder brethren of the faculty of physic, they now endeavoured to imitate them, and sought after new methods of increasing their sordid gains. This they effected by treacherously and surreptitiously invading the provinces of physicians and chirurgeons, and alluring the populace under the specious pretence of giving advice in physic and chirurgery *gratis*; which fatal delusion readily insnared the ignorant vulgar, who could not be sensible of their gross ignorance and manifest incapacity for such an undertaking, nor apprehend that these very bountiful gentry took care to tax their medicines with their invaluable advice; so that their prescriptions (of which, it must be confessed, they are always most liberal,) are now charged infinitely more than their real value, or what those of physicians and chirurgeons (though of more intrinsic worth, being certainly better adapted to particular exigencies, to the constitution, and the indications of cure,) might be made up and sold for. And thus, by iniquitously enhancing the prices of remedies, and giving them in unnecessary abundance, they clandestinely acquired unmerited fees, equal to those justly due to the most regularly educated and most experienced physicians or chirurgeons.

"That this is the present case, every intelligent, candid apothecary must confess. And these fallacies might be further evinced beyond dispute, were the generality to be made sensible of the just rates of medicines, and the most monstrous, extravagant bills they are frequently charged. To see two drachms of sal prunel, not worth a penny, disguised with some insignificant colour and an unintelligible pompous title, and sold for sixteen pence, must surely move your contempt and indignation!

"I have known a gentleman's bill, who sickened on Monday and died the Wednesday following, amount to above five pounds, though his careful apothecary had but a street's breadth to cross between his shop and the patient's lodgings. It is more than probable that, if the unhappy sufferer took to the amount of his bill in medicines, it was the cause of his death, and would, though he had the most athletic constitution; but, to acquit his apothecary of this *murder*, every one of the profession must confess that no man could well require or consume medicines to that value in the time. Then, what physician could expect so much for his attendance in the ordinary manner for so long?

"Sure there is nothing more inconsistent with common reason, than the taking of apothecaries from their proper office and station! I never saw a patient that was not desirous the apothecary should himself prepare whatsoever medicines should be prescribed for him; and if he is employed in *quacking*, and his time devoted to that alone, how is it possible he can see his customers justly served?

"It is commonly said, 'that physicians and apothecaries kill more than they cure.' However ludicrous or satirical this phrase may sound, it is a melancholy consideration that the ill-natured sarcasm often proves just, and is likely to continue so, until the apothecaries and druggists are brought under some proper regulations, which may confine them within the just limits of their respective callings. For, into such an abyss of depravity and degeneracy are these men fallen, by their grasping at matters beyond their sphere, that now (through ignorance or dishonesty) there is scarce a medicine of any worth to be had genuine."

As the book of Dr. Lucas is now, I should suppose, not easily to be procured, I have made sufficiently ample extracts from it to enable Mr. Dickinson to form some notion of the state of the profession in Lucas's time. What effect its publication then produced, I do not exactly know; but, when Lucas afterwards became a physician and a member of Parliament, he introduced a Bill for the reform of abuses in pharmacy, which is in force to this day, and known by the name of Lucas's Act.

To all this Mr. D. may rejoin, "The state of pharmacy in Ireland eighty years ago, has no reference to the present time: such abuses may have existed formerly, but it is quite impossible for any thing of the kind now to occur." I answer, that it appeared in evidence on a late trial between two apothecaries in Dublin, that calomel was dispensed by *guess*, without being weighed!—that laudanum was kept in a bottle which was labelled *pure water!*—it was sworn by an *apothecary*, that he believed "a man has a better chance of his life by not taking medicines at all!"—and it was also proved, that one of the parties asserted he was nearly poisoned by a medicine prepared in his own shop! Is it necessary for me to offer another observation on this subject? If Mr. Dickinson still doubts, would he believe "though one were to rise from the dead?"

Mr. Dickinson may, notwithstanding, still endeavour to support his opinions on the principle of "expediency," and of the "impracticability" of altering the present system. To this I answer, that, where the apothecaries are confined to the sole business of compounding medicines, such restriction is found by experience to be both "expedient" and "practicable." On the continent, in general, apothecaries are not permitted to practise as physicians or surgeons; and this prohibition has not been observed to occasion any public inconvenience. In France, according to the present regulations, no person can be admitted a practising apothecary who has not completed his twenty-fifth year, and passed eight years in the establishment of a regularly licensed apothecary. The examinations are three in number; two on the theory, and the third on the practice, of pharmacy. The last continues for four days, during which time nine chemical or pharmaceutical experiments are performed by the candidate.

Two professors of the School of Medicine, accompanied by two members of the School of Pharmacy, and an officer of police, once a year at the least, visit the shops and warehouses of apothecaries and druggists, to ascertain the quality of their medicines: such as are badly prepared or adulterated are seized, and legal proceedings instituted against the offender.

Apothecaries cannot give or sell any medicinal preparations, or compound drugs, except according to the prescriptions of physicians or surgeons, or by the written directions of the officers of health given under their hands. They are prevented from selling any secret medicines, and are obliged, in preparing those necessary to be kept in their shops, to conform to the directions of the Dispensatories authorized by the Schools of Medicine.

Before I conclude, I wish to advert to the last paragraph in Mr. Dickinson's letter, in which he says, "I concur in your plan, either of lowering the terms of your attendance, or of shutting out the incompetent practitioner by supplying your patients with medicines. Notwithstanding my concurrence in the propriety of your proposal, which to me seems well calculated to meet the exigencies of society, I cannot so easily reconcile the inconsistency of your argument against the union of medicine with pharmacy, when I find that, while you affirm the incompatibility of the two offices in one person, in order to exclude the apothecary from prescribing, your proposition goes near to make the physician a pharmaceutist, to prevent the apothecary from assuming the duties of the physician."

To this charge of inconsistency, I have not rendered myself liable; for, to the subject of physicians supplying their patients with medicines, I only cursorily alluded; and then merely observed, that, if apothecaries continued to neglect their shops, it might become a matter for consideration, whether physicians, from a regard to their patient's safety, ought not to supply them with pure and accurately compounded medicines. I never proposed, or even hinted at, the propriety of physicians keeping shops, or compounding the recipes of other physicians. I know that, in the treatment of disease, a few simple and cheap medicines, judiciously administered, are sufficient for every useful purpose; and I suggested that physicians might find it necessary to supply such medicines, without charging for them: not that I decidedly approved of the plan, but because I conceived it to be an effectual method of counteracting the system at present pursued by apothecaries.

*York-street, Dublin; 18th December,* 1820.

